# TatBC, TatB, and TatC form structurally autonomous units within the twin arginine protein transport system of *Escherichia coli*

**DOI:** 10.1016/j.febslet.2007.07.044

**Published:** 2007-08-21

**Authors:** George L. Orriss, Michael J. Tarry, Bérengère Ize, Frank Sargent, Susan M. Lea, Tracy Palmer, Ben C. Berks

**Affiliations:** a Department of Biochemistry, University of Oxford, South Parks Road, Oxford OX1 3QU, United Kingdom; b Department of Molecular Microbiology, The John Innes Centre, Norwich NR4 7UH, United Kingdom; c School of Biological Sciences, University of East Anglia, Norwich NR4 7TJ, United Kingdom; d Sir William Dunn School of Pathology, University of Oxford, South Parks Road, Oxford OX1 3RE, United Kingdom

**Keywords:** BN-PAGE, blue native-polyacrylamide gel electrophoresis, Twin arginine, Tat, Protein transport, Blue native-PAGE, Membrane proteins

## Abstract

The Tat (twin arginine translocation) system transports folded proteins across bacterial and thylakoid membranes. The integral membrane proteins TatA, TatB, and TatC are the essential components of the Tat pathway in *Escherichia coli*. We demonstrate that formation of a stable complex between TatB and TatC does not require TatA or other Tat components. We show that the TatB and TatC proteins are each able to a form stable, defined, homomultimeric complexes. These we suggest correspond to structural subcomplexes within the parental TatBC complex. We infer that TatC forms a core to the TatBC complex on to which TatB assembles.

## 1 Introduction

The Tat (twin arginine translocation) system transports folded proteins across the cytoplasmic membrane of prokaryotes and the thylakoid membrane of plant chloroplasts [1–4]. The Tat pathway is involved in a wide range of cellular functions including biosynthesis of respiratory and photosynthetic electron transfer chains, formation of the bacterial cell envelope, bacterial motility, establishing of the nitrogen-fixing symbiosis, quorum sensing, and bacterial pathogenesis [5,6]. The task faced by the Tat system is mechanistically challenging because it involves transporting large protein substrates of differing size and surface properties across a membrane while maintaining the membrane permeability barrier.

In the bacterium *Escherichia coli* the Tat system is minimally composed of the three integral membrane proteins TatA, TatB, and TatC [7–10]. TatC is a polytopic membrane protein, whereas TatA and TatB are sequence-related proteins comprising an N-terminal membrane-anchoring α-helix followed at the cytoplasmic side of the membrane by a basic amphipathic α-helix and then a water-soluble region of random coil structure. Despite their sequence similarities genetic analysis has shown that TatA and TatB have discrete roles in the *E. coli* Tat pathway [8,9]. TatB and TatC form a high molecular weight complex containing multiple copies of each of the constituent subunits [11]. This TatBC complex acts as the initial membrane binding site for Tat substrates [12,13] and then recruits TatA to form the active translocation site [13,14]. TatA forms homo-oligomeric ring-like structures that are likely to constitute the protein translocating channels of the Tat system [15].

In spite of the clear functional separation between TatA and TatBC the influence of TatA on the structure and assembly of the TatBC complex is still unclear. When purified from a strain overproducing all of TatA, TatB, and TatC the TatBC complex contains a proportion of the TatA protein present in the bacterium [11,16]. Similarly it has been reported that at native levels of Tat protein expression TatA can be co-immunoprecipitated with TatB if TatC is also present suggesting that the three proteins form a complex [11]. It has further been reported that the TatBC complex is unstable in the absence of TatA [17]. Taken together these observations have suggested that TatA has an obligate and important structural role within the TatBC complex. Nevertheless, in recent affinity tagging experiments using Tat proteins expressed at native levels we were unable to detect an association between TatA and the TatBC complex [18]. This observation has prompted us to re-examine the involvement of TatA in the formation and structure of the TatBC complex. We find that TatA is not required for the assembly or stability of the TatBC complex.

We have probed the structural organisation of the TatBC complex by separately producing and characterizing TatB and TatC. It had previously been reported that TatC is highly unstable in the absence of TatB [9]. However, we find that both TatB and TatC are each able to form a stable, defined, homomultimeric complex. We suggest that these species correspond to structural subcomplexes within the TatBC complex. In particular, we infer that TatC forms a single core subcomplex onto which TatB is assembled.

## 2 Materials and methods

### 2.1 Strains and plasmids

All expression constructs used in this study are based on vectors from the pQE series (Qiagen, Crawley, United Kingdom). Plasmid pFAT586 [19] produces TatB with a C-terminal hexa-histidine tag. Plasmid pFAT75CH [16] produces TatABCDE with a hexa-histidine tag on the C-terminus of the TatC protein. Plasmid pFAT75CHΔA [20] produces TatBC with a hexa-histidine tag on the C-terminus of the TatC protein. Plasmid pFAT588 produces TatC with a hexa-histidine tag on the C-terminus. To construct pFAT588 the *tatC* gene was amplified from the chromosomal DNA of *E. coli* strain MC4100 [21] using the primers 5′-GCGCCCATGGTGTCTGTAGAAGATACTCAACCGC-3′ and TATCH2 [16]. The resulting amplicon was digested with NcoI and BglII and cloned into the same sites in pQE60 (Qiagen). Plasmid pFAT588C4A produces a TatC variant in which the four cysteine residues of the native protein have been substituted with alanine residues and which has a hexa-histidine tag on the C-terminus of the protein. This plasmid was constructed in the same way as pFAT588 except that plasmid pUNITATCC4H [22] was used as the PCR template.

C43ΔTat(DE3) is a derivative of strain C43(DE3) [23] in which the *tatABC* genes have been replaced with the apramycin resistance cassette of plasmid pIJ773 [24]. The strain was constructed by the lambda Red recombinase method of Datsenko and Wanner [25] using the primers TatA1 and TatD1 [8] with BW25113 Δ*tatABC*::*Apra* [22] as the template.

### 2.2 Production of Tat proteins and preparation of membrane fractions

Strains were co-transformed with the appropriate expression plasmid and pREP4 (Kan^R^, *lacI*^+^, Roche Molecular Biochemicals) and cultured aerobically at 37 °C in LB medium [26]. TatB_His_, TatABC_His_DE, and TatBC_His_ were produced in *E. coli* strain DADE (MC4100 Δ*tatABCD*Δ*tatE*) [27]. The cells were grown at 37 °C. When the culture reached an OD_600_ of 0.4–0.5 IPTG was added to a final concentration of 2 mM. Growth was allowed to continue at 37 °C for a further 5–6 h before harvesting by centrifugation for 15 min at 7000 × *g*. The TatC_His_ variants were produced in either strain C43(DE3) or strain C43Δtat (DE3). The cells were cultured at 30 °C until they reached an OD_600_ of 0.4–0.5. IPTG was then added to a final concentration of 0.4 mM and growth continued for 18 h at 25 °C before harvesting.

Pelleted cells were resuspended in 20 mM MOPS–HCl, pH7.2, 200 mM NaCl (bufferA) supplemented with 10 μg ml^−1^ DNaseI (Sigma–Aldrich, Gillingham, United Kingdom) 50 μg ml^−1^ lysozyme (Sigma–Aldrich, Gillingham, United Kingdom), and a Complete Mini-EDTA protease inhibitor cocktail tablet (Roche Molecular Biochemicals, Lewes, United Kingdom). The cells were broken in a French Press and a crude membrane fraction was isolated as described previously [16].

### 2.3 Solubilization and purification of Tat complexes

The TatBC_His_-containing membrane fraction was solubilized at a protein concentration of 5 mg ml^−1^ for 1 h at 4 °C in buffer A containing 2% (w/v) digitonin (Merck Biosciences, Nottingham, United Kingdom). Unsolubilized material was removed by centrifugation at 257 000 × *g* for 30 min at 4 °C. The resulting supernatant was applied to a Ni(II)-loaded HiTrap Chelating HP column (5 ml; GE Healthcare, Amersham, United Kingdom) that had been pre-equilibrated with buffer A containing 0.1% digitonin and 50 mM imidazole. The column was further washed with 25 ml buffer A containing 0.1% digitonin and 120 mM imidazole, before elution of the protein with a 120–700 mM imidazole gradient over 20 ml. Fractions containing purified TatBC were identified by SDS–PAGE and Coomassie Brilliant Blue G-250 staining. The purest fractions were pooled and concentrated to 500 μl using an Amicon Ultra 4 concentration device (Millipore Corporation, Bedford, MA, USA) with a 100 kDa cut-off. In the final step, the concentrated sample was either applied to a HR10/30 Superose 6 (HP) or a Superdex 200 (HR) size exclusion column (both GE Healthcare, Amersham, United Kingdom) equilibrated in 20 mM MOPS–HCl pH 7.2, 200 mM NaCl and 0.1% digitonin.

TatC_His_-containing membrane fractions were solubilized at a protein concentration of 10 mg ml^−1^ for 2 h at 4 °C in buffer A containing 2% (w/v) digitonin. Unsolubilized material was removed by centrifugation at 257 000 × *g* for 30 min at 4 °C. The supernatant material was applied to a Ni(II)-loaded HiTrap Chelating HP column (5 ml) that had been pre-equilibrated with buffer A containing 0.1% digitonin and 25 mM imidazole. The column was further washed with 25 ml buffer A containing 0.1% digitonin and 75 mM imidazole, before elution of the protein with a 75 mM to 500 mM imidazole gradient over 20 ml. Fractions containing purified TatC were identified by SDS–PAGE, pooled, concentrated and subject to size exclusion chromatography as detailed for TatBC_His_.

### 2.4 Protein analysis methods

Blue native-polyacrylamide gel electrophoresis (BN-PAGE) was performed under the standard conditions described by Schägger and von Jagow [28]. SDS–PAGE and immunoblotting were performed as described [29,30]. Immunoreactive bands were visualized with the ECL system (GE Healthcare, Amersham, United Kingdom). The antibodies used were an anti-TatB serum raised against gel-eluted TatB protein and an anti-pentahistidine-horse radish peroxidase conjugate (Qiagen, Lewes, United Kingdom). Protein concentrations were determined by the D_C_ protein assay (Bio-Rad, Hercules, USA).

## 3 Results

### 3.1 TatA has no significant structural role in the TatBC complex

There have been conflicting reports as to the presence of TatA as a component of the *E. coli* TatBC complex and of the necessity of this subunit for the structural integrity of the complex [11,16–18]. To directly address this issue we have produced TatB and TatC in the absence of TatA or any other Tat components. TatC was provided with a C-terminal hexa-histidine tag to allow subsequent purification studies. This tagging strategy has previously been shown not to interfere with Tat function [16].

**Fig. 1:**
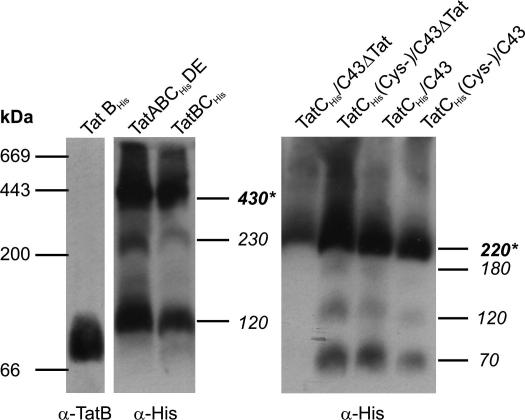
BN-PAGE analysis of Tat complexes in digitonin solubilized membranes. Whole membrane fractions were prepared and solubilized in digitonin as detailed in the methods section, subjected to BN-PAGE in a 5–18% polyacrylamide gradient gel, and then immunoblotted with either anti-TatB (left hand lane) or anti-His tag (all other lanes) sera. Membranes were prepared from cells overproducing the indicated Tat proteins. The background strains used were the Δ*tatABCD*Δ*tatE* strain DADE (two left hand panels), the Tat wildtype strain C43(DE3) or its Δ*tatABC* derivative C43ΔTat(DE3). Soluble extract from 50 μg of membrane protein was loaded in each lane. The migration positions of the standard proteins thyroglobulin (669 kDa), ferritin (443 kDa), β-amylase (200 kDa) and bovine serum albumin (66 kDa) are indicated at the left of the figure. The immunologically detected TatC_His_-containing components are labelled with their apparent molecular weight in kDa at the right of each panel and with the most abundant species marked with an asterisk.

We found that TatB and TatC_His_ were both present in the TatA-free cells (data not shown) indicating that TatA is not absolutely required for the stability of TatB or TatC. To ascertain whether the absence of TatA affects the ability of TatB and TatC_His_ to form stable complexes we used blue native-PAGE (BN-PAGE) to analyse the TatC_His_-containing complexes found in membrane extracts solubilized with the detergent digitonin (Fig. 1). Digitonin is known to maintain the integrity of the TatBC complex and has been used in all previous BN-PAGE analysis of the TatBC complex [31,32]. When TatA is present the TatC_His_ protein is found in prominent complexes with apparent molecular weights of 430 kDa and 120 kDa as well as a low abundance complex of 230 kDa. The same species, with the same pattern of abundances, were observed in membrane extracts from cells expressing only TatB and TatC_His_. Thus, TatA is not required for TatC to form distinct multiprotein complexes, nor does it detectably affect the form of these complexes.

**Fig. 2:**
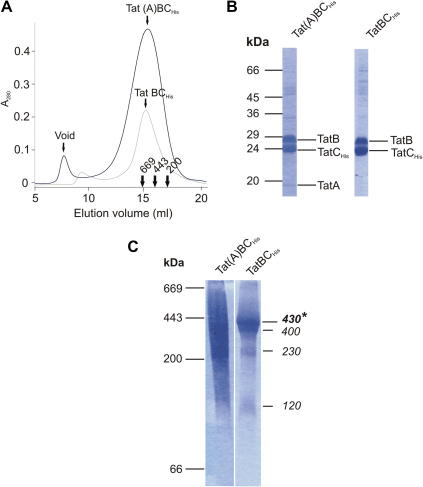
Characterization of the purified Tat(A)BC_His_ and TatBC_His_ complexes. Membranes were prepared from strain DADE, solubilized in digitonin and the TatC_His_-containing complexes purified by Ni(II) affinity chromatography. (A) Size exclusion chromatography of the affinity-purified complexes on a Superose 6 (HP) column. The absorbance of the column eluant at 280 nm (A_280_) is plotted for each complex. The elution positions of the standard proteins thyroglobulin (669 kDa), ferritin (443 kDa) and β-amylase (200 kDa) are indicated. (B) SDS–PAGE analysis of the purified complexes. The peak fraction from each of the Superose 6 column separations shown in (A) was subjected to SDS–PAGE. Proteins were visualized by Coomassie Brilliant Blue staining. The molecular masses in kDa of standard proteins are given on the left of the figure. The Tat subunits are identified to the right of each panel. (C) BN-PAGE analysis of the purified complexes. The samples used are the same as those analysed in (B). A 3–15% polyacrylamide gradient was employed and 10 μg protein was loaded in each lane. Following electrophoresis the gel was stained with Coomassie Brilliant Blue. The migration positions of standard proteins (as in Fig. 1) are indicated to the left of the figure. The apparent molecular weights in kDa of the Tat complexes are indicated to the right of the figure with the most abundant species identified with an asterisk.

To characterize the observed TatC_His_-containing complexes in more detail the complexes were purified by Ni(II) affinity chromatography. The TatC_His_-containing complexes were then further purified by size exclusion chromatography. When TatC_His_ was co-expressed with both TatA and TatB the purified TatC-containing complexes eluted from the size exclusion column in a relatively broad peak, corresponding to an apparent molecular weight of around 600 kDa (Fig. 2A), that contained TatA, TatB, and TatC_His_ proteins (Fig. 2B). This preparation is essentially identical to that reported previously by another group [11]. For consistency with the earlier literature we will term this material Tat(A)BC_His_ to indicate that only a small proportion of the total TatA present in the cell co-purifies with TatBC. When TatC_His_ was co-produced with TatB, but not TatA or other Tat components, the purified TatC_His_-containing complexes eluted at the same position on the size exclusion column as Tat(A)BC_His_ (Fig. 2A). SDS–PAGE analysis of the peak fraction from the size exclusion column confirmed that the purified complexes contain TatB and TatC_His_ but no TatA (Fig. 2B). The purified Tat(A)BC_His_ and TatBC_His_ complexes were further analysed by BN-PAGE (Fig. 2C). Purified Tat(A)BC was poorly resolved (Fig. 2C) possibly indicating an increase in the heterogeneity of the complexes upon purification. By contrast, the TatBC_His_ complex gave the same species with apparent molecular weights of 430 kDa, 230 kDa, and 120 kDa (Fig. 2C) that had been observed in the soluble extract by immunoblotting (Fig. 1). In addition, the increased resolution obtained with the purified material showed a fourth species of apparent molecular mass 400 kDa running slightly ahead of the 430 kDa species (and see also Fig. 2C). The more quantitative detection afforded by Coomassie staining relative to immunoblotting shows that the 430 kDa species is overwhelmingly the most abundant species present (and see also Fig. 2C).

**Fig. 3:**
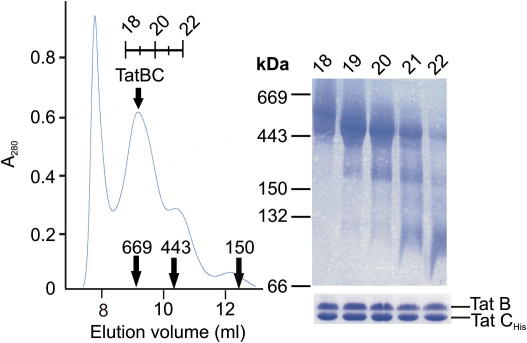
The TatBC_His_species identified by BN-PAGE can be separated by size exclusion chromatography. Digitonin-solubilized and Ni(II) affinity-purified TatBC_His_ was subject to size exclusion chromatography on a Superdex 200 (HR) column. The absorbance of the column eluant at 280 nm (*A*_280_) is plotted in the left hand panel. The elution positions of thyroglobulin (669 kDa), ferritin (443 kDa) and alcohol dehydrogenase (150 kDa) are indicated, as are the elution positions of fractions 18–22. The right hand panel shows a BN-PAGE analysis of the indicated column fractions. A 3–15% polyacrylamide gradient was employed. The migration positions of standard proteins (as in Fig. 1) are shown to the left of the panel. The panel under the BN-PAGE gel shows an SDS–PAGE analysis of the same column fractions. Proteins on both the BN-PAGE and SDS–PAGE gels were visualized by Coomassie Brilliant Blue staining.

The conditions used in BN-PAGE are known to disrupt some detergent-solubilized membrane protein complexes. We, therefore, investigated whether the low abundance TatC_His_-containing species observed by BN-PAGE could be an artefact of this electrophoretic method. The affinity-purified TatBC_His_ complex was chromatographed on a size exclusion column possessing a lower molecular weight fractionation range than in the earlier experiment and successive fractions of the eluted protein peak were analyzed by BN-PAGE (Fig. 3). The size exclusion column was able to partially resolve the species that migrate at different apparent molecular weights on BN-PAGE. This demonstrates that the species observed by BN-PAGE correspond to complexes of different molecular size and are not an artefact of the analytical method.

### 3.2 TatC forms a distinct multimeric complex

We attempted to probe the structural organisation of the TatBC complex by separately producing the constituent TatB and TatC polypeptides. We found that a hexa-histidine-tagged version of TatB could be successfully overproduced and membrane targeted in the absence of any other Tat components. Following solubilization with digitonin this TatB_His_ protein ran as a single band on BN-PAGE with an apparent molecular mass of 80 kDa (Fig. 1, left hand panel). TatB therefore forms a specific low order oligomer when TatC is not present. Since TatB has a protomer molecular weight of 18.4 kDa, and BN-PAGE tends to overestimate native molecular weights [33], the TatB species observed here is not more than a homotetramer and most probably smaller. TatB dimers have previously been identified in membranes by chemical crosslinking [19] and TatB tetramers have been detected in TatBC complexes by site-specific disulfide crosslinking [22].

**Fig. 4:**
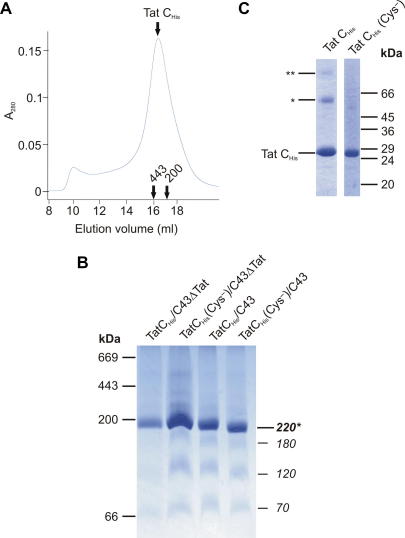
Characterization of purified TatC_His_ complexes. (A) Size exclusion chromatography of the TatC_His_ protein on a Superose 6 (HR) column. The TatC_His_ sample analyzed had been produced in the Tat wild-type strain C43(DE3), solubilized with digitonin, and purified by Ni(II) affinity chromatography. The absorbance of the column eluant at 280 nm (*A*_280_) is plotted. The elution positions of standard proteins are indicated, as is the peak of the TatC_His_ protein identified by SDS–PAGE analysis of the column fractions. (B) BN-PAGE analysis of purified TatC_His_ complexes. TatC_His_, or its cysteineless variant TatC_His_(Cys^−^), were produced either in the Tat wild-type strain C43(DE3) or its Δ*tatABC* derivative C43ΔTat(DE3). All complexes were solubilized in digitonin and purified by successive Ni(II) affinity chromatography and size exclusion chromatography steps. A 4–16% polyacrylamide gradient was employed and 9 μg protein was loaded in each lane. Following electrophoresis the gel was stained with Coomassie Brilliant Blue. To the left of the figure are shown the migration positions of standard proteins (as in Fig. 1). The apparent molecular weights in kDa of the TatC complexes are indicated to the right of the figure with the most abundant species identified with an asterisk. (C) SDS–PAGE was used to analyse (left hand lane) the purified TatC_His_ complex from the size exclusion column shown in (A) and (right hand lane) the cysteineless variant TatC_His_(Cys^−^) purified in the same way. Proteins were visualized by Coomassie Brilliant Blue staining. The migration positions of the TatC_His_ monomer (TatC_His_), and of TatC_His_ dimers (∗) and trimers (∗∗) are indicated to the left of the panel.

We attempted to overproduce TatC in the absence of other Tat components by expressing a hexa-histidine-tagged version of the protein in strain DADE which lacks all *tat* genes. Only low level TatC_His_ production was obtained. We did, however, observe that TatC_His_ could be produced to high levels in the *tat* wild-type strain C43(DE3) in which the other Tat components are present only at native levels. When solubilized in digitonin the TatC_His_ protein was predominantly found in a complex of apparent molecular mass 220 kDa (Fig. 1, right hand panel). Lower abundance species of 180 kDa, 120 kDa, and 70 kDa were also observed. The detergent solubilized TatC_His_ protein was purified by Ni(II)-affinity chromatography followed by size exclusion chromatography. The elution position of TatC_His_ from the size exclusion column corresponded to an apparent native molecular weight of 400 kDa (Fig. 4A). Purified TatC_His_ gave the same banding pattern in BN-PAGE as it had in the original detergent extract with the 220 kDa species confirmed as overwhelmingly the most abundant species present (Fig. 4B). Since the TatC protomer has a molecular weight of 28.9 kDa this suggests that TatC forms a distinct major oligomer, probably corresponding to a heptamer or smaller.

SDS–PAGE analysis of purified TatC_His_ (Fig. 4C) showed not only the expected band for TatC at 27 kDa but also higher molecular weight species which immunoblotting confirmed to also contain TatC_His_ and which would correspond to TatC_His_ dimers and trimers. The abundance of the higher order TatC_His_ oligomers observed in the SDS–PAGE gel decreased when the samples were treated with reductant or if TatC_His_ was purified in the presence of DTT. This suggested that the oligomers arose, at least in part, from disulfide linkages between TatC protomers. The higher order TatC_His_ bands were not observed by immunoblotting crude membranes suggesting that the disulfide links form during protein solubilization and purification.

We considered it possible that the low abundance TatC_His_ species observed by BN-PAGE (Figs. 1 and 4B) corresponded to complexes containing the disulfide-linked TatC_His_ molecules. To investigate this possibility we produced and purified a TatC_His_ variant in which the four native cysteine residues have been replaced with alanines. This variant has previously been shown to support normal Tat transport activity [34]. The purified cysteineless TatC_His_ showed only the monomeric TatC_His_ species on SDS–PAGE (Fig. 4C) confirming that the higher order oligomers arise from disulfide linkages between TatC protomers. The banding pattern on BN-PAGE was, however, indistinguishable from that of the cysteine-containing protein (Fig. 1 right hand panel, Fig. 4B). Thus the minor BN-PAGE species do not correlate with disulfide links between protomers. It is notable that the TatBC_His_ preparation does not contain disulfide-linked TatC molecules (Fig. 2B) suggesting that the TatB protomers shield the reactive TatC cysteine(s) to some extent.

The TatC_His_ complex had been purified from a strain containing native levels of the other Tat components. While there was clearly no stoichiometric co-purification of TatB or TatA with the TatC_His_ complex it remained a possibility that these other Tat components had some catalytic role in the assembly of TatC_His_. To address this possibility we constructed a derivative of strain C43(DE3) containing an in-frame deletion of *tatABC* and then produced TatC_His_ and cysteineless TatC_His_ in this background. BN-PAGE analysis of digitionin-solubilized membrane extracts identified the same TatC_His_ complexes as those found in the Tat^+^ parental strain with a most abundant species of apparent molecular weight 220 kDa (Fig. 1 right hand panel). This analysis was substantiated when the TatC_His_ and cysteineless TatC_His_ complexes produced in the *tatABC* deletion background were purified and analysed by BN-PAGE (Fig. 4C). We conclude that the TatC protein is able to form a distinct, stable, multimeric complex independent of TatA and TatB.

## 4 Discussion

Within the *E. coli* Tat system the complex formed between the TatB and TatC proteins forms a major functional unit. A number of studies have reported that the TatA protein is able to interact with this TatBC complex [11,13,16,17]. However, we have now used expression and purification studies to show that TatA is not required for either TatBC complex assembly or stability (Figs. 1 and 2). In the absence of TatA we find that the predominant digitionin-solubilized TatBC species has an apparent molecular weight of 430 kDa as assessed by BN-PAGE (Figs. 1 and 2C). Co-production of TatBC with TatA did not cause any significant change in the mobility of this complex (Fig. 1) even though some TatA now co-purifies with TatBC (Fig. 2C). Previous BN-PAGE studies of strains producing the full Tat system identified the major digitonin-solubilized TatBC-containing species as a complex with an apparent molecular weight of either 440 kDa [32] or 370 kDa [31] which is in broad agreement with the data presented here.

We found that TatC was able to form a distinct oligomeric complex when produced in the absence of other Tat components (Figs. 1 and 4). This contrasts with a previous study that reported that TatC is highly unstable in the absence of its TatB partner [9]. TatB and TatC occur at an equimolar ratio in the TatBC complex [11]. TatC should, therefore, provide 60% of the mass of the TatBC complex. In good agreement with this we observe that the apparent molecular weight of the digitonin-solubilized TatC complex on BN-PAGE (220 kDa) is approximately 50% of the apparent molecular weight of the TatBC complex determined in the same way (430 kDa). This implies that the TatC complex corresponds to the entire TatC component of the full TatBC complex. We infer that the TatC protomers form an autonomous substructure within the TatBC complex and that interactions between TatC molecules are sufficient for the assembly of this substructure. Although it has previously been reported that the transmembrane helix of TatB is not required for the formation of high molecular weight TatBC complexes [17] whether this engineered material formed a distinct complex, what its molecular weight was, and whether it was stabilized by the extramembranous domains of TatB were not addressed.

The TatB component of the TatBC complex could also be produced independently of other Tat proteins. It also forms a single oligomeric species when solubilized in digitonin (Fig. 1). This suggests that TatB, like TatC, forms autonomous oligomeric substructures within the TatBC complex, an inference that is in agreement with previous crosslinking studies that show direct interactions between TatB protomers [19,22]. However, the TatB oligomer has an apparent molecular weight of less than 100 kDa by BN-PAGE. It is, therefore, likely that more than one copy of the TatB oligomer will be present in each TatBC complex. These TatB subdomains would be linked to each other only indirectly via binding to the TatC core. This is most easily envisaged if the TatB units bind peripherally to the TatC core rather than being located on the interior of the complex as previously tentatively inferred from crosslinking studies [22].

In summary, we have demonstrated that the TatBC complex does not require TatA or other Tat components for its assembly, or stability and that TatC forms a distinct, multimeric species in the absence of its TatB partner. These observations resolve a number of conflicts in the Tat literature. Our data suggest that TatC forms a stable core within the TatBC complex upon which the TatB component assembles.
